# Stroboscopic light stimulation as a controllable probe of the psilocybin state: a mechanistic framework and a falsifiable prediction

**DOI:** 10.3389/fphar.2026.1852799

**Published:** 2026-09-30

**Authors:** Kacper Żełabowski, Tomasz Wieczorek, Adam Hamed

**Affiliations:** 1 Scientific Society for Psychopharmacology, Department of Forensic Medicine, Wroclaw Medical University, Wrocław, Poland; 2 Interdisciplinary Society for Prevention of Lifestyle Diseases, Wroclaw, Poland; 3 Laboratory of Spatial Memory, Nencki Institute of Experimental Biology, Polish Academy of Sciences, Warsaw, Poland

**Keywords:** 5D-ASC, 5-HT_2A_ receptor, altered states of consciousness, bottom-up cortical processing, predictive coding, psilocybin, stroboscopic light stimulation

## Abstract

Stroboscopic light stimulation (SLS) and psilocybin each induce altered states whose phenomenology and neurophysiology partly overlap, including posterior alpha-band changes and visual imagery. The two states engage the alpha band through different operations - pharmacological suppression under psilocybin and frequency-selective visual drive under SLS. We formalize their possible interaction for prediction and falsification. The framework proposes that, by relaxing the precision weighting of high-level cortical priors and, through 5-HT_2A_ activation, suppressing posterior alpha-band power, psilocybin creates a state in which an exogenous, frequency-controlled visual rhythm can modulate the perceiver’s experience. The thesis concerns precision rather than response amplitude. Because existing neuroimaging comes mostly from eyes-closed rest, where directional evidence is contested, the falsification does not depend on which sensory-inclusive model, Relaxed Beliefs Under Psychedelics (REBUS) or cortico-striatal-thalamo-cortical (CSTC), is correct. The framework predicts that, within a prespecified near-peak psilocin window, rhythmic SLS raises the Visionary Restructuralization dimension of the five-dimensional Altered States of Consciousness questionnaire (5D-ASC) relative to a luminance- and rate-matched arrhythmic control - an increase a precision framework predicts even if evoked amplitude does not rise. A complementary, report-independent endpoint, the intermodulation response to two-frequency stimulation, is proposed. State-dependence is tested as the drug-by-rhythmicity interaction in a 2 × 3 factorial design (placebo or psilocybin × rhythmic SLS, arrhythmic control, or no stimulation), with the sign of any interaction evaluated on a pre-declared scale. An adequately precise negative interaction would count against the central state-dependence prediction for the tested conditions. The framework organizes five axes around this prediction: titration of experiential intensity at fixed dose, content shaping through frequency specificity, modulation of difficult-experience phenomenology, testing whether resting-state signatures depend on sensory context, and individual-difference profiling of SLS responsiveness. The framework makes no claim of therapeutic enhancement. Its aim is mechanistic and methodological.

## 1 Introduction

Recent years have seen renewed clinical and mechanistic research on serotonergic psychedelics. Controlled trials evaluate psilocybin in the context of Major Depressive Disorder (MDD) (Carhart-Harris et al., 2021), in distress related to cancer (Griffiths et al., 2016), and in alcohol misuse (Bogenschutz et al., 2022). The acute psilocybin state is heterogeneous both within the individual and across the population. Set, setting, and dose shape the quality of the experience (Studerus et al., 2012; Studerus et al., 2011). Evidence from LSD and drug-free flicker suggests that sensory qualities may form an additional modulating axis (Kaelen et al., 2015; Montgomery et al., 2024). Compared with music or interpersonal context, stroboscopic light has received less systematic study as a candidate perceptual modulator.

Kaelen et al. demonstrated that lysergic acid diethylamide (LSD) intensifies the emotional reaction to music (Kaelen et al., 2015), and music-listening is a consistent feature of psychedelic-therapy sessions (Kaelen et al., 2018). Montgomery et al. showed that flickering light stimulation also strengthens the emotional response to music in healthy participants who had not received a psychedelic (Montgomery et al., 2024), extending the result to a stimulus that is itself a candidate perceptual modulator. Both publications present these perceptual stimuli as modulators of affect, raising the question of what happens when a visual stimulus of fixed frequency meets a pharmacologically altered cortex. Stroboscopic light stimulation induces altered states of consciousness (ASC) with characteristic perceptual and neurophysiological correlates (Schwartzman et al., 2019; Amaya et al., 2023a; Hewitt et al., 2025). SLS sessions have been described in commercial contexts (Lumenate Nova, 2026), but the combined use of SLS and psilocybin has not been formalized. This paper proposes a mechanistic, falsifiable framework combining SLS and psilocybin, while prior work has drawn phenomenological parallels between the two states (Montgomery et al., 2024; Schwartzman et al., 2019).

The psychedelic state is widely proposed to change the relative precision, or weighting, of high-level priors against incoming sensory signals (Carhart-Harris and Friston, 2019; Stoliker et al., 2022; Alamia et al., 2020), although the directionality of this shift is contested (Section 3.3). Periodic visual stimulation elicits frequency-dependent cortical responses (Herrmann, 2001; Notbohm et al., 2016). The framework rests mainly on the Relaxed Beliefs Under Psychedelics (REBUS) model (Carhart-Harris and Friston, 2019), yet it is also neutral toward competing accounts of the psychedelic state and notes plainly where the bottom-up reading is contested. The paper does not propose SLS combined with psilocybin as a treatment for any psychiatric disorder. The framework is mechanistic and methodological, and its aim is to motivate experimental work on the interaction of a visual modulator with a predefined pharmacological state.

## 2 Scope and falsification

The REBUS model (Carhart-Harris and Friston, 2019) and the cortico-striatal-thalamo-cortical (CSTC) account reviewed by Doss et al. (2022) each admit a role for sensory drive in shaping the acute experience. The cortico-claustro-cortical (CCC) account, by contrast, centers on cognitive control rather than sensory binding. The framework’s predictions are testable regardless of which of these accounts proves correct.

Stoliker et al. applied dynamic causal modelling to resting-state functional magnetic resonance imaging (fMRI) with eyes closed in 24 participants receiving 0.2 mg/kg psilocybin. The connectivity analysis included 20 participants after head-motion thresholding. They found increased self-inhibition in the early visual area (EVA), the fusiform gyrus, the intraparietal sulcus, and the inferior frontal gyrus. They stated that their results do not support the bottom-up assumption of the REBUS model between visual and associative regions in the resting state (Stoliker et al., 2025).

The framework restricts its claim to the neurophysiological state characteristic of SLS and does not extend it to the resting state. It holds that the precision weighting of cortical signalling is altered under psilocybin in a way that becomes measurable specifically under SLS, when an exogenous rhythm provides frequency-selective visual drive within a band whose endogenous power is pharmacologically suppressed. On the precision account, this proposed susceptibility follows from the reduced precision of high-level priors rather than from a necessary increase in stimulus-evoked amplitude. Its existence and sign remain empirical questions. Most psychedelic resting-state fMRI datasets have used eyes-closed acquisition (McCulloch et al., 2022). The prediction does not require that the amplitude of stimulus-evoked responses increase. The active variable is the weighting of, and oscillatory engagement with, the sensory stream, not its magnitude. The term entrainment is reserved here for phase-based or individual-alpha-frequency-dependent evidence (Notbohm et al., 2016). The prediction remains falsifiable regardless of which of the sensory-inclusive accounts (REBUS or CSTC) proves correct.

If SLS delivered within a prespecified near-peak psilocin window does not raise the Visionary Restructuralization parameter in the 5D-ASC relative to a control condition (matched arrhythmic light, with the same luminance as in the test condition and the same mean flash rate), and an adequately powered study with successful manipulation checks estimates the drug-by-rhythmicity interaction precisely enough to exclude the prespecified effect of interest, the central state-dependence prediction would not be supported. An imprecise null would remain inconclusive. A positive drug-by-rhythmicity interaction, by contrast, would support a state-dependent rise in Visionary Restructuralization but would not by itself fix the direction of cortical flow, which is adjudicated by the directional endpoints set out in Section 5.4. The primary prediction is embedded in a factorial design that sharpens its interpretation: the pharmacological condition (placebo or psilocybin) is crossed with three stimulus conditions, rhythmic SLS, an arrhythmic control, and a no-stimulation condition, so that the scientifically central claim, that the effect of rhythmic structure is conditional on the pharmacologically altered cortex, is carried by the drug-by-rhythmicity interaction. Within this design the luminance contrast (light versus dark) estimates sensory/light drive while the rhythmicity contrast estimates the contribution of temporal regularity. The latter does not by itself establish entrainment.

If the primary prediction holds but no other dimension of the 5D-ASC shows SLS-dependent modulation under the tested placement, the scope of the framework narrows to a single effect in the visual domain, without the broader phenomenological relevance that the framework expects.

The framework specifies the direction of the result but not effect-size thresholds. Those thresholds belong to a later, pre-registered protocol that would translate the predictions into a statistically powered design. The present work concerns whether the predictions are coherent and mechanistically verifiable.

## 3 Mechanistic substrate

### 3.1 5-HT_2A_ agonism and the role of cortical signalling

The receptor-level action of classic psychedelics is well documented. Aghajanian and Marek showed that 5-HT_2A_ activation potentiates the late, non-synchronous components of glutamatergic excitatory postsynaptic potentials on the apical dendrites of layer V cortical pyramidal cells. They proposed that the perceptual and affective changes induced by hallucinogens follow from altered glutamatergic transmission in the cortex (Aghajanian and Marek, 1999). Vollenweider and Kometer set this finding within a wider neurobiological context, framing 5-HT_2A_ agonism and the resulting increase in cortical glutamatergic activity as the primary pharmacological mechanism of the psychedelic state (Vollenweider and Kometer, 2010). Stoliker et al. extended the analysis of receptor pharmacology to the cortical hierarchy (Stoliker et al., 2022). Although 5-HT_2A_ agonism is the documented mechanistic basis of the rehierarchization, psilocin has a broader binding profile (Rickli et al., 2016), with substantial, sub-micromolar affinity for the 5-HT_2C_ and 5-HT_1A_ receptors (Ki approximately 0.094 and 0.123 μM, against approximately 0.049 μM at 5-HT_2A_). This matters because the two relevant receptors need not act in the same direction: 5-HT_1A_ activation is generally inhibitory and hyperpolarizes cortical pyramidal cells, so it can act in partial opposition to 5-HT_2A_-driven excitation (Carhart-Harris and Nutt, 2017; Nichols, 2016). The net effect on cortical excitability, and hence on susceptibility to an exogenous visual drive, need not be a simple monotonic function of psilocin concentration. The framework therefore does not treat findings from different 5-HT_2A_ agonists as pharmacologically interchangeable.

### 3.2 The altered hierarchy and the empirical record

Related cortical signatures have been reported across chemical compounds and recording modalities, but their strength and direction are not uniform. Muthukumaraswamy et al. observed broadband cortical desynchronization in magnetoencephalography after psilocybin intake, with the most marked decreases in the posterior association cortices, including the posterior cingulate cortex (PCC) (Muthukumaraswamy et al., 2013). Task-free fMRI after intravenous psilocybin showed decreased cerebral blood flow and blood-oxygen-level-dependent (BOLD) signal in medial hub regions and reduced medial prefrontal cortex (mPFC)-PCC coupling (Carhart-Harris et al., 2012). The arterial spin labeling (ASL) scans used a fixation cross, whereas the separate BOLD scans were acquired with eyes closed. A recent review identified recurring within- and between-network connectivity changes while emphasizing substantial methodological heterogeneity (Farré-Colomés et al., 2026). Schartner et al. found an increase in Lempel-Ziv signal diversity under ketamine, LSD, and psilocybin, identifying complexity as a cross-compound marker of the psychedelic state. For psilocybin specifically the increase did not reach statistical significance on any single measure (for the single-channel measure LZsN 86% of participants showed higher diversity), whereas for ketamine and LSD the core diversity measures (ACE, LZc, LZs, LZsN) survived Bonferroni correction (Schartner et al., 2017). Kometer et al. showed that 5-HT_2A_ activation reduces alpha-band oscillations and the N170 visual potential, with the N170 reduction correlating with Visionary Restructuralization (Kometer et al., 2013). In a simultaneous electroencephalography (EEG)-fMRI study, Timmermann et al. found that N,N-dimethyltryptamine (DMT) increased global functional connectivity (GFC) alongside network disintegration and desegregation. The spatial map of the GFC-intensity relationship overlapped with an independent positron emission tomography (PET)-derived map of 5-HT_2A_ receptor density, which the authors read as implying that 5-HT_2A_ signalling underlies these effects and that they likely generalize to other 5-HT_2A_ agonist psychedelics (Timmermann et al., 2023).

### 3.3 Direction of the effect under stimulation: precision rather than amplitude

The framework’s main mechanistic assumption is that the psychedelic state alters the balance of signal transmission in the cortex, which becomes evident during stimulation with a rhythmic exogenous stimulus. The closest directional evidence comes from studies of cortical travelling waves under DMT at eyes-closed rest, not from combined psilocybin and SLS.

Alamia et al. showed that the top-down alpha waves typical of rest with eyes closed underwent a marked reduction after DMT administration, whereas bottom-up signals were strengthened. These results were interpreted as confirmation of the hypothesis that psychedelics relax the precision weighting of priors, shifting the relative balance of cortical information flow (Alamia et al., 2020). On a precision account this rebalancing concerns weighting, not amplitude: a relaxation of high-level priors lets ascending signals propagate with greater latitude without implying that their evoked amplitude grows. This distinction is decisive, because the available evidence indicates that 5-HT_2A_ agonists do not simply amplify stimulus-evoked responses and in several components reduce them: psilocybin lowers the N170 (Kometer et al., 2013), a hallucinogenic 5-HT_2A_ agonist reduces visual response gain in mouse V1 (Michaiel et al., 2019), serotonergic input exerts a separable, partly suppressive gain control over evoked activity in mouse visual cortex (Azimi et al., 2020), and in mouse auditory cortex psilocybin produces a biphasic change in responsiveness (Brockett and Francis, 2024). A naive reading, in which psilocybin amplifies the cortical response to an exogenous rhythm, is therefore not supported. A rhythm delivered to a cortex whose endogenous alpha has been pharmacologically suppressed may shape experience without a net increase in the magnitude of the evoked response.

As noted in Section 2, Stoliker et al. showed increased self-inhibition and reduced inhibitory top-down connectivity between visual and associative regions after psilocybin administration during eyes-closed rest. The authors concluded that their data do not support the bottom-up principle of the REBUS model under these conditions (Stoliker et al., 2025). Independent work on LSD cautions against an overly literal reading of how psychedelics redistribute cortical information flow. Preller et al. found that LSD does not produce the undifferentiated cortical inundation predicted by the CSTC model, but instead increases information flow to particular cortical regions (Preller et al., 2019). Doss et al., in turn, report evidence from directional modelling for intensified top-down transmission from the parahippocampal gyrus to the visual cortex, contrary to the REBUS model’s account of the parahippocampal gyrus as an originator of enhanced bottom-up flow (Doss et al., 2022). These results do not refute the concept under discussion, but rather specify its limits.

Existing data do not allow the direction of information flow to be attributed unambiguously to the condition studied. The results of Alamia et al. (indicating bottom-up flow) and of Stoliker et al. (suggesting top-down flow and self-inhibition) were obtained in rest with eyes closed - after DMT and after psilocybin respectively. The psilocybin result came from a restricted dynamic-causal-modelling analysis of four regions of interest and should not be generalized to the resting-state literature as a whole (Stoliker et al., 2025). The state not yet examined under psilocybin is the eyes-closed state under stimulation, in which an external rhythmic stimulus competes with endogenous dynamics (Figure 1). A recent book chapter by Godfrey et al. notes that limited research on functional connectivity and cortical travelling waves suggests that directed top-down control may decrease while bottom-up signalling may increase, potentially indicating a transient reversal of typical hierarchical organization, and notes that replication is warranted (Godfrey et al., 2025).

**FIGURE 1:**
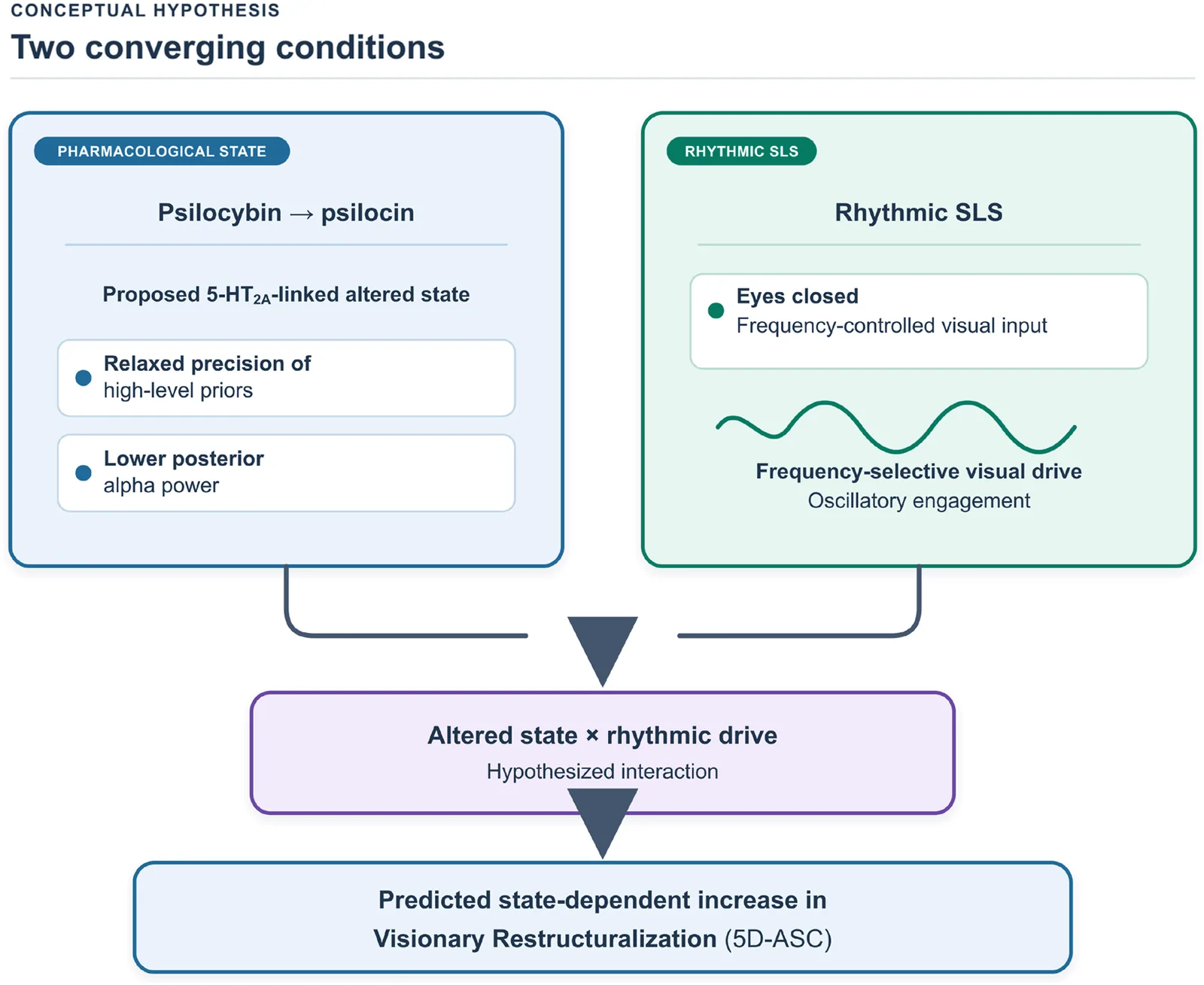
Hypothesized mechanism linking psilocybin and rhythmic stroboscopic light stimulation (SLS). Psilocin-associated 5-HT_2A_ activation is proposed to relax the precision-weighting of high-level priors and reduce posterior alpha-band power. Under eyes-closed rhythmic stimulation, frequency-selective visual drive is proposed to engage this altered state and increase Visionary Restructuralization. The diagram presents a mechanistic hypothesis and does not, by itself, establish entrainment of endogenous oscillations.

The cross-compound evidence is consistent with a reweighting in favour of sensory drive without amounting to undifferentiated flooding. Under LSD, Carhart-Harris et al. reported expanded resting functional connectivity of primary visual cortex (V1), correlated with complex imagery at r = 0.74, with uncorrected p = 0.0016 and Bonferroni-corrected *p* = 0.006 (Carhart-Harris et al., 2016). The convergent yet indirect nature of this support is itself a limitation, developed in Section 8. The evidence for travelling-wave direction comes from DMT, the evidence for expanded visual connectivity from LSD, and direct resting-state fMRI evidence exists for psilocybin. None of these establishes travelling-wave direction under combined psilocybin and SLS.

### 3.4 Pharmacokinetic background

According to the review by Dinis-Oliveira, psilocin becomes detectable in plasma within 20–40 min of oral psilocybin administration and subjective effects subside after 4–6 h (Dinis-Oliveira, 2017). In a direct oral-dose study, median psilocin Tmax was approximately 2.0 h and its elimination half-life was 3 h (SD 1.1). Parent psilocybin was not detected, so these data do not support assigning it an exact half-life (Brown et al., 2017). The intensity of subjective experience correlates with both plasma psilocin concentration and 5-HT_2A_ receptor occupancy, the latter being related to plasma concentration according to a single-site binding model (Madsen et al., 2019). Because individual time-concentration curves vary, the framework situates the test within a prespecified near-peak window rather than at a universal T_max_.

## 4 Phenomenological modulation

### 4.1 Stroboscopic phenomenology alone

SLS on its own, without psilocybin administration, induces an ASC. Schwartzman et al. reported an increase in EEG signal diversity during SLS, accompanied by subjective reports of altered perception. In a between-study comparison, the authors found that ASC profiles under SLS resembled, in several respects, those reported for the psilocybin state (Schwartzman et al., 2019). Amaya et al. showed that the frequency of flicker systematically shapes the geometry of induced hallucinations, and that rhythmic stimulation yields more structured imagery than arrhythmic stimulation matched for mean frequency (Amaya et al., 2023a). The proposed arrhythmic control is light matched for luminance and mean flash rate (the mean number of flashes per second), lacking rhythmic structure. Rhythmic 10 Hz flicker reliably induces transient visual hallucinations, whereas an arrhythmic sequence delivering the same visual energy produces fewer of them, which makes rhythmicity rather than light itself the relevant contrast and justifies the matched arrhythmic control (Amaya et al., 2025). Imaging during flicker shows engagement beyond primary cortex: functional connectivity increases between the lateral geniculate nucleus, early visual areas, and proximal upstream visual areas (Amaya et al., 2023b), while rhythmic 10-Hz flicker increases connectivity between ventroanterior thalamic nuclei and higher-order visual cortices, with the latter associated with subjective intensity (Amaya et al., 2025). Hewitt’s recent review provides a comprehensive account of the phenomenology and neurophysiology of stroboscopically induced hallucinations (Hewitt et al., 2025). Notbohm et al. set the cortical response to periodic light against the accounts of entrainment and superposition, finding phase coupling strongest when stimulation approached individual alpha frequency (Notbohm et al., 2016). Genuine entrainment of endogenous oscillators is best documented in the alpha band. Accordingly, the present model does not assume that every flicker-evoked steady-state visual evoked potential (SSVEP) response reflects entrainment of an internal rhythm, and reserves the term for phase-based or individual-alpha-frequency-dependent evidence.

Hartman’s 1963 study of mescaline, LSD, and psilocybin with a flicker apparatus is sometimes cited as a precursor of this line of research (Hartman and Hollister, 1963). The apparatus was an episcotister, which produces flicker with the eyes open rather than stroboscopic stimulation with the eyes closed, and the dependent measure was color perception. The study establishes that periodic visual input “interacts” with classic psychedelics, but it is not a direct precedent for eyes-closed SLS protocols.

### 4.2 Sensory modulation of the psychedelic state

The interaction of sensory stimuli with the psychedelic state has already been documented empirically in the auditory domain. Kaelen et al. observed that LSD intensifies the emotional reaction to music (Kaelen et al., 2015). Montgomery et al. showed that flickering light (tested at 10 Hz), in healthy drug-free participants, likewise strengthens this response, supporting the potential of visual stimuli as independent, non-pharmacological modulators (Montgomery et al., 2024). Preller and Vollenweider described the multidimensional structure of the psychedelic experience and its dynamics, creating a conceptual frame that allows predictions to be formulated for its individual components (Preller and Vollenweider, 2018). These data show that the emotional response to a sensory stimulus can be enhanced both pharmacologically (LSD) and non-pharmacologically (flicker). A candidate neural mechanism for this affective modulation is the prefrontal alpha/delta switch. Frequency-specific cortical drive is not affectively neutral, with alpha-band activity linked to hedonic states and long-term potentiation and delta-band activity to aversive states and long-term depotentiation, such that closed-loop alpha-frequency stimulation can shift affective valence (Burgdorf and Moskal, 2023). This raises the possibility that the flicker rhythm itself carries affective valence rather than merely amplifying an existing emotional response - though that evidence derives from electrical stimulation of the medial prefrontal cortex in rodents, where the relevant band is 6–7 Hz, so its transfer to occipital visual flicker in humans remains indirect. Whether stimulus parameters such as modality and flicker frequency selectively engage specific dimensions of the experience, rather than scaling it uniformly, is precisely what the framework’s directional prediction is built to test.

### 4.3 Predicted phenomenological signatures

The model formulates predictions for each of the five dimensions of the 5D-ASC questionnaire, yet only one of them is a falsifying test for the framework. For Visionary Restructuralization the prediction is directional and primary: SLS within a prespecified near-peak psilocin window should raise the scores on this dimension (Dittrich, 1998; Studerus et al., 2010) relative to a control condition (arrhythmic light of the same luminance and mean flash frequency). The sign of the interaction is treated as an empirical result rather than an assumption and must be evaluated on a pre-declared scale. The falsifying test is therefore the drug-by-rhythmicity interaction - not the main effect of stimulation, which the SLS-alone literature already predicts. Its adequately precise failure would count against the central prediction for the tested conditions, provided that data quality and manipulation checks are adequate (Section 2). An imprecise null would remain inconclusive.

The remaining dimensions are treated as secondary and exploratory. For Auditory Alterations no directional change is predicted. For Dread of Ego Dissolution no direction of SLS-related change is assumed, because difficult experience can occur across set-and-setting conditions (Studerus et al., 2012). For the Oceanic Boundlessness dimension no directional modulation is predicted. For the Vigilance Reduction dimension no particular direction of change is assumed either. Drug-free flicker can affect broader ASC phenomenology, particularly Vigilance Reduction. However, differences between 3- and 10-Hz conditions did not survive correction across domains (Bartossek et al., 2021). In all cases the outcomes are secondary and exploratory, and the null hypothesis is not adopted by default.

Mortaheb et al. observed that the recurrent pattern of hyperconnectivity characterizing the resting state after psilocybin intake is associated primarily with the Oceanic Boundlessness dimension and only secondarily with Visionary Restructuralization (Mortaheb et al., 2024). Even so, the present model adopts Visionary Restructuralization as the primary outcome. This rests on two premises. First, Visionary Restructuralization is the 5D-ASC dimension specific to the visual modality and therefore the dimension most directly amenable to modulation by SLS. Second, it is the only 5D-ASC dimension with a direct empirical link both to the visual signature of psilocybin - the N170 reduction correlates specifically with Visionary Restructuralization (Kometer et al., 2013) - and to the effect of flicker, which on its own induces visual-perceptual phenomenology in the absence of any drug (Amaya et al., 2023a; Bartossek et al., 2021). The pattern described by Mortaheb et al. refers to a resting-state signature, whereas the assumptions of the present model concern stimulation-driven phenomenological changes (Mortaheb et al., 2024).

### 4.4 Operationalization

The 5D-ASC, with the dimensions Visionary Restructuralization, Auditory Alterations, Oceanic Boundlessness, Dread of Ego Dissolution, and Vigilance Reduction, is proposed as the primary phenomenological instrument (Dittrich, 1998; Studerus et al., 2010). The eleven lower-order Altered States of Consciousness scales (11-ASC) (Studerus et al., 2010) and the Mystical Experience Questionnaire (MEQ-30) (Maclean et al., 2012) are retained as exploratory secondary instruments. The brief MEQ-4 and Challenging Experience Questionnaire (CEQ-7), validated by Strickland et al., correlate with their full counterparts at r = 0.89 and r = 0.90. Both were validated on retrospective recall of naturalistic psychedelic users with therapeutic intent (Strickland et al., 2024). Within the present model, the methodologically optimal study design compares SLS with an arrhythmic control matched for luminance and mean flash frequency and includes an appropriate washout period between sessions. The development of a detailed procedural protocol remains a matter for future empirical research. The minimal factorial test is shown in Figure 2.

**FIGURE 2:**
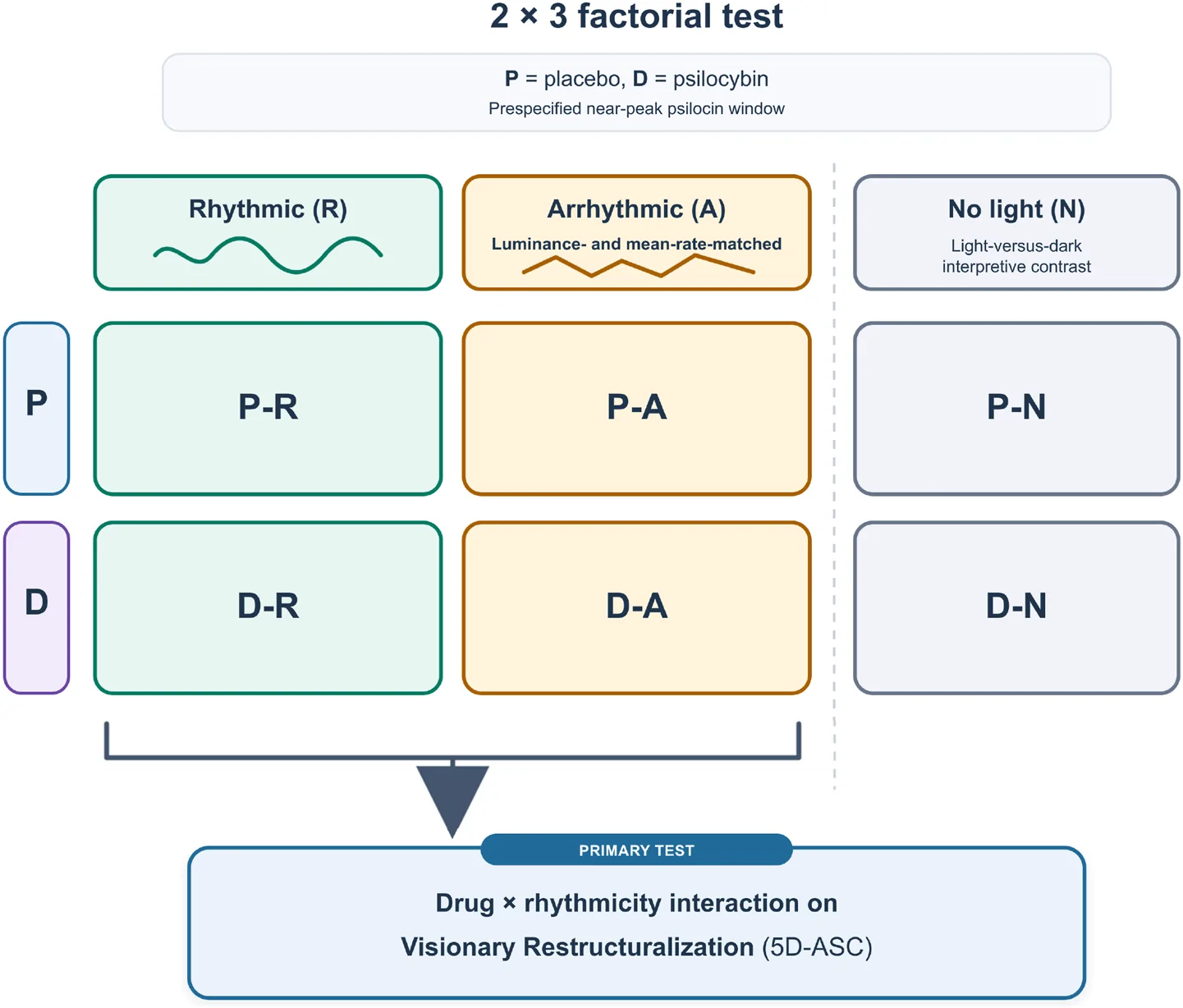
Minimal experimental test of the state-dependence hypothesis. A 2 × 3 factorial design crosses drug (placebo or psilocybin) with stimulus (rhythmic SLS, luminance- and mean-rate-matched arrhythmic light, or no stimulation) within a prespecified near-peak psilocin window. The primary confirmatory test is the drug-by-rhythmicity interaction on Visionary Restructuralization. The light-versus-dark contrast estimates nonspecific sensory drive. Neurophysiological measures are complementary mechanistic readouts and do not replace the phenomenological interaction.

## 5 Research use-cases

The framework organizes the research programme around five scenarios. They are formulated as research questions with measurable endpoints. The scenarios are listed separately because they differ methodologically.

### 5.1 Titration of the intensity of the experience

This scenario asks whether SLS can reversibly modulate the intensity of the experience at a drug dose set by protocol. Dose titration in psilocybin research is limited by the irreversibility of oral administration and by the slow pharmacokinetics of psilocin relative to the dynamics of any interventions during the session (Dinis-Oliveira, 2017). By contrast, SLS parameters can be modified on a timescale of seconds, and stimulation can be stopped completely.

The fundamental research question concerns whether the variables of the SLS protocol (frequency, luminance, and duty cycle) show, under such conditions, a measurable stimulus-response relationship with the Visionary Restructuralization dimension of the 5D-ASC questionnaire. A non-pharmacological manipulation would allow researchers to test reversible modulation of a state initiated by a fixed oral psilocybin dose. Because SLS is reversible on this timescale, the scenario also asks whether the response to the same stimulation may differ across the rising, peak, and falling phases of the psilocin concentration curve. We hypothesize that differing affinities at 5-HT_1A_ and 5-HT_2A_ could make their relative contributions concentration-dependent, so maximal susceptibility to SLS may not coincide with peak psilocin concentration (Rickli et al., 2016; Carhart-Harris and Nutt, 2017; Nichols, 2016).

### 5.2 Shaping of content

This scenario exploits the frequency specificity of stroboscopic stimulation. Different flash frequencies elicit frequency-specific resonance in the visual cortex (Herrmann, 2001), and varied temporal profiles of stimulation (frequency and rhythmicity) induce different phenomenological states (Amaya et al., 2023a). A formal substrate connects the two manipulations at the level of geometry: the Klüver form constants are planforms that arise from spontaneous symmetry-breaking in primary visual cortex, and the same neural-field modelling identifies flickering light and hallucinogens as triggers of this common instability (Bressloff et al., 2001; Billock and Tsou, 2012). On this account psilocybin and SLS converge on a shared pattern-forming substrate, not because 5-HT_2A_ agonism uniformly increases evoked gain, but through altered cortical excitability that unmasks spontaneous pattern formation. This yields content predictions beyond classification: the distribution of reported form-constant classes should shift under psilocybin, and the spatial structure of the percept should be biased by flicker frequency, quantifiable against the form-constant taxonomy after the retinocortical (log-polar) mapping (Bressloff et al., 2001; Billock and Tsou, 2012).

The model assumes that within the content of the psilocybin experience one can distinguish a pharmacological baseline and a component shaped directly by sensory stimuli. This hypothesis is subject to verification through a comparison of results across the individual scales of the 5D-ASC and 11-ASC for different variants of SLS, as well as through a structured classification of the visual content reported by participants.

### 5.3 Modulation of the dimensions of difficult experience

Applying an SLS protocol during a controlled assessment may lead to shifts, undetermined in direction at this stage, in Challenging Experience Questionnaire scores (Strickland et al., 2024). We also propose examining Dread of Ego Dissolution without assuming a direction of change. It should not be used as an intervention during acute distress. Pre-session mindset already explains a modest but significant part of the variance in challenging-experience scores under controlled administration of the substance (Angyus et al., 2024), which provides a reference point for evaluating any changes induced by stimulation. Such a result would justify further research on perceptual-pharmacological interactions. The alpha/delta-valence finding is retained as a possible affective confound, not as evidence that alpha-frequency SLS would reduce difficult experience in humans (Burgdorf and Moskal, 2023). Any study would require predefined stopping rules and continuous safety monitoring.

### 5.4 Methodological control

SLS is an experimentally controlled perceptual variable, allowing researchers to separate the pharmacological effects from the effects of the drug’s interaction with a sensory stimulus. In most neuroimaging studies of psychedelics, the eyes-closed state is treated as a resting condition. However, this state is a specific sensory context in which the visual system, although cut off from external stimuli, shows its own internal generative activity. As shown in Section 3.3, it is partly under these conditions that the evidence for bottom-up directional connectivity raises the greatest doubts (Stoliker et al., 2025).

Applying an SLS protocol allows this hidden confound to be turned into an explicit experimental variable. This rests on the assumption that psilocybin shifts the balance between intrinsic and stimulus-driven cortical activity, and preclinical work supports it. In awake mouse sensory cortex psilocybin changed the amplitude of stimulus-evoked responses and raised functional connectivity while leaving stimulus selectivity intact, a pattern the authors interpret as a reweighting of intrinsic against stimulus-driven influence that parallels observations reported in visual cortex (Brockett and Francis, 2024). The fundamental research question concerns the degree to which the cortical signature of the psychedelic state - measured in the conventional resting-state paradigm - is determined by the sensory context itself (Alamia et al., 2020; Muthukumaraswamy et al., 2013; Schartner et al., 2017). The framework proposes the following optimal endpoints: analysis of signal complexity (the Lempel-Ziv algorithm), parieto-occipital alpha-band power, and the directionality of travelling waves in the parieto-occipital cortex. These parameters should be assessed comparatively between the SLS condition and an arrhythmic control precisely matched for luminance and mean flash rate. This issue is of key importance: the mechanistic models on which later postulates rest are credible only to the degree that the baseline adopted for their measurement is sound. Beyond these, the amplitude of the stimulus-evoked response is itself diagnostic: a precision account predicts that Visionary Restructuralization can rise under SLS even when evoked amplitude does not, whereas an amplified-bottom-up account predicts that the two move together. A complementary, report-independent endpoint follows from the nonlinearity of cortical processing: driving the visual system at two distinct frequencies f1 and f2 produces intermodulation components at their linear combinations (f1 + f2, f1 - f2, 2f1 - f2, and so on), which arise from nonlinear integration of the two inputs and index that integration (Gordon et al., 2019; Gundlach and Müller, 2013). This measure does not depend on the participant’s report, but it is not necessarily immune to attention or expectancy effects. Its direction and feasibility under psilocybin are not established, and it does not by itself identify the anatomical locus or direction of the interaction. Intermodulation should therefore complement, rather than determine, the mechanistic interpretation. It cannot uniquely distinguish relaxed priors, cortical gain, or thalamic gating. No study in the cited literature reports intermodulation responses under a psychedelic, so the endpoint remains a genuine novelty and an open question of feasibility. These contrasts are supported by the two-by-three design of Section 4.4. An interaction contrast of this kind may be harder to detect than a main effect, so any future test of the framework could be correspondingly demanding in scale, a constraint sharpened by photosensitivity exclusion and the cost of psilocybin sessions.

### 5.5 Profiling of individual responders

The response to psychedelics shows considerable inter-individual variability. An important issue is whether reactivity to SLS is an independent, measurable differentiating variable that interacts with the response to psilocybin. Angyus et al. (2024) validated the Imperial Psychedelic Predictor Scale as a prospective instrument, noting, however, its modest predictive power under naturalistic conditions. Moujaes et al. (2024), in turn, studying the effect of ketamine, demonstrated the multidimensionality of its neuronal and behavioural effects. For ketamine, they emphasized substantial individual variability, in which individualized response signatures emerged from group-level data. Group-averaged results may mask structured patterns of response in individual participants. Baseline individual alpha frequency and trait Absorption should therefore be examined as candidate continuous moderators of SLS responsiveness (Notbohm et al., 2016) and (Bartossek et al., 2021), without adding conditions to the factorial design. Whether psilocybin changes phase locking to flicker remains an open empirical question. It is also reasonable to examine whether brief pre-session phenotyping using SLS alone, correlated with a prospective predictor scale, allows the combined response profile to an SLS-with-psilocybin procedure to be predicted. Verification of this assumption would bring tangible methodological value, enabling finer participant-level profiling.

## 6 Safety considerations

### 6.1 Safety of light stimulation

Stroboscopic stimulation carries a documented risk of light-induced seizure activity in susceptible individuals (Hewitt et al., 2025). Screening can be adapted from the European clinical intermittent photic stimulation (IPS) methodology to include a personal and family history of photosensitive epilepsy and a photic-stimulation trial during an EEG examination (Kasteleijn-Nolst Trenité et al., 2012). Exclusion should be based on individual risk and screening findings rather than demographic group alone. The updated Epilepsy Foundation review (Fisher et al., 2022) is a useful additional reference here, and it raises a tension worth stating explicitly: eye closure is itself a provocative condition and sustained eyes-closed viewing is not protective, yet the eyes-closed state is central to the framework as formulated. Any screen should therefore use a multi-frequency procedure across the standard eye conditions rather than a single-frequency challenge.

The European photic-stimulation algorithm indicates that susceptibility spans a broad frequency range, with most patients sensitive between 10 and 30 Hz, and identifies eye closure as the most provocative test condition (Kasteleijn-Nolst Trenité et al., 2012). Harding and Jeavons report peak sensitivity at 15–20 flashes/s and photoparoxysmal responses to patterns oscillating at 10–20 Hz (Harding and Jeavons, 1994). Stimulation at a fixed, single frequency may also fail to detect a meaningful minority of photosensitivity cases. In unpublished personal data from 70 already photosensitive patients with generalized photoparoxysmal responses, testing only 18 Hz would have missed about 15% because their sensitivity occurred at other frequencies (Kasteleijn-Nolst Trenité et al., 2012). This is not a population estimate of photosensitivity or seizure risk. The alpha band employed here overlaps the sensitivity range and is therefore not free of seizure risk. The pre-inclusion provocation screen is an important safeguard, but it has not been validated as a guarantee of safety for combined SLS and psilocybin.

### 6.2 Psychedelic-specific safety

The proper reference point for an adverse psychological response is the characterization by Marrocu et al., who, in a self-selected naturalistic cohort using mixed psychedelics, found that 16.4% exceeded a one-standard-error-of-measurement decline threshold in psychological wellbeing at 4 weeks. Among the 16 participants who self-reported a prior personality-disorder diagnosis, 5 (31.2%) were negative responders (Marrocu et al., 2024). These observations are not controlled psilocybin event rates and do not estimate risk specific to SLS. Protocols combining SLS with psilocybin should retain the exclusion criteria rationalized in previous psilocybin studies and add the photic-stimulation criteria from Section 6.1. The cardiovascular effects characterized in the meta-analysis by Romeo et al. support retaining cardiovascular monitoring during the acute session (Romeo et al., 2024).

### 6.3 Interaction considerations

Whether combining SLS with psilocybin carries an interaction-specific risk beyond the additive risk of the two components remains an open empirical question. The cited literature provides no direct data on proconvulsant risk specific to combined SLS and psilocybin (Kasteleijn-Nolst Trenité et al., 2012), (Fisher et al., 2022), or (Romeo et al., 2024). Interaction-specific risk therefore remains unknown. We propose characterizing the potential interaction early using predefined stimulation parameters and a procedure-stopping protocol, while physiological signals and EEG are recorded continuously throughout the SLS phase. Any further research should be conditional on an initial safety-characterization stage designed to detect or characterise a predefined warning signal. Such a stage cannot exclude rare risk, and interaction-specific risk remains unknown.

### 6.4 Practical safeguards

Control over the stimulation, including the ability to reduce or stop it, should remain with the participant whenever they are capable of exercising it. We propose supervision by staff trained to manage participants during acute psychedelic effects. Because motor and verbal responses may be impaired at the culmination of the experience, the supervising staff must also be able to stop the stimulation independently. As an author-proposed safeguard, monitoring during the SLS phase would include continuous EEG with predefined stopping criteria for photoparoxysmal or epileptiform activity, measurement of heart rate and arterial pressure, and continuous qualitative observation. We further propose retaining the post-session care used in psilocybin research, including extended observation, psychological support, and integration of the experience.

## 7 Comparative context

SLS belongs to a broader group of non-pharmacological neuromodulation methods, and its position relative to alternative paradigms can be summarized. Repetitive transcranial magnetic stimulation (rTMS) is supported by formal evidence-based therapeutic guidelines for specified clinical indications (Lefaucheur et al., 2020). The mechanism of TMS rests on an externally induced, focal depolarization of the cerebral cortex, which distinguishes this method from SLS in terms of the transduction route and the equipment requirements.

The closest mechanistic analogue is transcranial alternating current stimulation (tACS). This is an electrical technique with a documented ability to entrain cortical oscillations in the alpha band (Helfrich et al., 2014). SLS rests on a partly analogous logic of synchronization, but the signal is transmitted by the visual route through the retina and the lateral geniculate nucleus rather than directly transcranially. Flicker has been shown to modulate functional connectivity between the lateral geniculate nucleus and upstream visual areas in a frequency-dependent way (Amaya et al., 2023b). In the auditory domain, music plays an analogous conceptual role (Kaelen et al., 2015; Montgomery et al., 2024), which has a long history of use in psychedelic research. Music and SLS are not mutually exclusive. SLS here is a tool oriented toward a specific mechanism, mainly because its purely frequency-based structure allows parametric manipulation and oscillatory analysis within individual bands, which is hard to achieve with musical stimuli.

Magni et al. proposed a model of modulation using Virtual Reality (VR) within the predictive-coding context (Magni et al., 2023). These authors treat VR-induced hallucinations as a tool for modifying the balance between top-down predictions and the bottom-up signal. There is a conceptual convergence here with the paradigm under discussion, although VR delivers an immersive, content-rich stimulus, while lacking a strictly frequency-based structure.

## 8 Limitations

The strongest reference point for the altered-weighting account is the DMT travelling-wave research by Alamia et al. (2020). It is corroborated by the multimodal EEG-fMRI work of Timmermann et al. (2023). Although the authors of both publications extrapolate their results to other psychedelics that are 5-HT_2A_ receptor agonists, these analyses do not replace direct testing of travelling-wave direction under psilocybin. Direct resting-state fMRI evidence exists for psilocybin, but the directional claim remains untested during SLS. The extrapolation therefore runs along two axes at once, from DMT to psilocybin and from the eyes-closed rest in which the travelling-wave evidence was recorded to the stimulated regime in which the framework locates its prediction. Several human and animal findings indicate that psilocybin and related 5-HT_2A_ agonists modulate the amplitude of stimulus-evoked responses in a time- and component-dependent manner, psilocybin increasing the medial P1 while decreasing the N170 (Kometer et al., 2013) and, in mouse auditory cortex, producing a biphasic change (Brockett and Francis, 2024), rather than amplifying it uniformly. The framework accommodates this by locating the active variable in precision weighting and oscillatory engagement rather than in response amplitude.

Harari et al. (2025) showed restricted synergistic effects of psilocybin combined with 2.5 h of light exposure, rather than an SLS protocol, on visual-cortex gene expression in male mice. The possibility of translating these results to the human cerebral cortex remains an open question. Inter-individual generalization is equally unsettled (Section 5.5), so all directional predictions should at present be interpreted as assumptions about the group mean and further characterized with respect to individual variability.

Blinding is another significant methodological challenge that remains difficult to resolve fully. Effective masking of participants to rhythmic SLS versus the arrhythmic control is highly problematic, and expectancy effects on self-report measures, including the primary endpoint, will remain considerable. This is one reason the framework pairs the self-report contrast with a report-independent neurophysiological endpoint, the intermodulation response under two-frequency drive, while recognizing that this measure is not necessarily immune to attention or expectancy effects (Section 5.4).

The literature uses ‘flicker’ and ‘stroboscopic’ light stimulation with partial overlap. Intensity, waveform, and eye-condition differ across the studies synthesized here. The present framework treats them as one family while acknowledging that these parameter differences may constrain the primary prediction’s validity.

## 9 Conclusion

The framework hypothesizes that the state induced by psilocybin may be susceptible to exogenous perceptual variables. The character and direction of any such susceptibility remain open empirical questions. The central criterion is whether the drug-by-rhythmicity interaction supports a state-dependent increase in the Visionary Restructuralization dimension of the 5D-ASC within a prespecified near-peak psilocin window, using rhythmic SLS and the luminance- and mean-rate-matched arrhythmic control. If an adequately precise drug-by-rhythmicity result fails under successful manipulation and adequate data quality, it would count against the central state-dependence prediction for the tested conditions. An imprecise null would remain inconclusive. If the assumption is confirmed, protocols using psilocybin will gain a new independent variable, a frequency-controlled exogenous visual modulator manipulated independently of the pharmacological dose. Because the prediction is embedded in a factorial design, the central claim of state-dependence is carried by the drug-by-rhythmicity interaction rather than by either factor alone, and the combined state gains a complementary, report-independent measure of cortical nonlinear integration. This allows more precise conclusions about state-dependent processing and makes it possible to carry out the research projects outlined above. The next step is to conduct a pre-registered study of adequate statistical power, based on a predefined statistical analysis plan. The present work sets out the predictive frame of the model and the criteria for its falsification. Designing the experiment that verifies these assumptions is a task for the next stage of research.

## Data Availability

The original contributions presented in the study are included in the article/supplementary material, further inquiries can be directed to the corresponding author.

## References

[B1] Aghajanian G. K. Marek G. J. (1999). Serotonin and hallucinogens. Neuropsychopharmacology 21, 16S–23S. 10.1016/S0893-133X(98)00135-3 10432484

[B2] Alamia A. Timmermann C. Nutt D. J. VanRullen R. Carhart-Harris R. L. (2020). DMT alters cortical travelling waves. Elife 9, e59784. 10.7554/eLife.59784 33043883 PMC7577737

[B3] Amaya I. A. Behrens N. Schwartzman D. J. Hewitt T. Schmidt T. T. (2023a). Effect of frequency and rhythmicity on flicker light-induced hallucinatory phenomena. PLoS One 18, e0284271. 10.1371/journal.pone.0284271 37040392 PMC10089352

[B4] Amaya I. A. Schmidt M. E. Bartossek M. T. Kemmerer J. Kirilina E. Nierhaus T. (2023b). Flicker light stimulation induces thalamocortical hyperconnectivity with LGN and higher-order thalamic nuclei. Imaging Neurosci. 1, 1–20. 10.1162/imag_a_00033 40799697 PMC12007554

[B5] Amaya I. A. Nierhaus T. Schmidt T. T. (2025). Thalamocortical interactions reflecting the intensity of flicker light-induced visual hallucinatory phenomena. Netw. Neurosci. 9, 1–17. 10.1162/netn_a_00417 40161990 PMC11949548

[B6] Angyus M. Osborn S. Haijen E. Erritzoe D. Peill J. Lyons T. (2024). Validation of the imperial psychedelic predictor scale. Psychol. Med. 54, 3539–3547. 10.1017/S0033291724002204 39327922 PMC11496213

[B7] Azimi Z. Barzan R. Spoida K. Surdin T. Wollenweber P. Mark M. D. (2020). Separable gain control of ongoing and evoked activity in the visual cortex by serotonergic input. Elife 9, e53552. 10.7554/eLife.53552 32252889 PMC7138610

[B8] Bartossek M. T. Kemmerer J. Schmidt T. T. (2021). Altered states phenomena induced by visual flicker light stimulation. PLoS One 16, e0253779. 10.1371/journal.pone.0253779 34197510 PMC8248711

[B9] Billock V. A. Tsou B. H. (2012). Elementary visual hallucinations and their relationships to neural pattern-forming mechanisms. Psychol. Bull. 138, 744–774. 10.1037/a0027580 22448914

[B10] Bogenschutz M. P. Ross S. Bhatt S. Baron T. Forcehimes A. A. Laska E. (2022). Percentage of heavy drinking days following psilocybin-assisted psychotherapy vs placebo in the treatment of adult patients with alcohol use disorder: a randomized clinical trial. JAMA Psychiatry 79, 953–962. 10.1001/jamapsychiatry.2022.2096 PMC9403854 36001306

[B11] Bressloff P. C. Cowan J. D. Golubitsky M. Thomas P. J. Wiener M. C. (2001). Geometric visual hallucinations, Euclidean symmetry and the functional architecture of striate cortex. Philos. Trans. R. Soc. Lond B Biol. Sci. 356, 299–330. 10.1098/rstb.2000.0769 11316482 PMC1088430

[B12] Brockett A. T. Francis N. A. (2024). Psilocybin decreases neural responsiveness and increases functional connectivity while preserving pure-tone frequency selectivity in mouse auditory cortex. J. Neurophysiol. 132, 45–53. 10.1152/jn.00124.2024 38810366 PMC11383378

[B13] Brown R. T. Nicholas C. R. Cozzi N. V. Gassman M. C. Cooper K. M. Muller D. (2017). Pharmacokinetics of escalating doses of oral psilocybin in healthy adults. Clin. Pharmacokinet. 56, 1543–1554. 10.1007/s40262-017-0540-6 28353056

[B14] Burgdorf J. S. Moskal J. R. (2023). A prefrontal cortex alpha/delta switch controls the transition from positive to negative affective states. Discov. Ment. Health 3, 19. 10.1007/s44192-023-00044-3 37861869 PMC10564693

[B15] Carhart-Harris R. L. Friston K. J. (2019). REBUS and the anarchic brain: toward a unified model of the brain action of psychedelics. Pharmacol. Rev. 71, 316–344. 10.1124/pr.118.017160 31221820 PMC6588209

[B16] Carhart-Harris R. L. Nutt D. J. (2017). Serotonin and brain function: a tale of two receptors. J. Psychopharmacol. 31, 1091–1120. 10.1177/0269881117725915 28858536 PMC5606297

[B17] Carhart-Harris R. L. Erritzoe D. Williams T. Stone J. M. Reed L. J. Colasanti A. (2012). Neural correlates of the psychedelic state as determined by fMRI studies with psilocybin. Proc. Natl. Acad. Sci. U. S. A. 109, 2138–2143. 10.1073/pnas.1119598109 22308440 PMC3277566

[B18] Carhart-Harris R. L. Muthukumaraswamy S. Roseman L. Kaelen M. Droog W. Murphy K. (2016). Neural correlates of the LSD experience revealed by multimodal neuroimaging. Proc. Natl. Acad. Sci. U. S. A. 113, 4853–4858. 10.1073/pnas.1518377113 27071089 PMC4855588

[B19] Carhart-Harris R. Giribaldi B. Watts R. Baker-Jones M. Murphy-Beiner A. Murphy R. (2021). Trial of Psilocybin *versus* escitalopram for depression. N. Engl. J. Med. 384, 1402–1411. 10.1056/NEJMoa2032994 33852780

[B20] Dinis-Oliveira R. J. (2017). Metabolism of psilocybin and psilocin: clinical and forensic toxicological relevance. Drug Metab. Rev. 49, 84–91. 10.1080/03602532.2016.1278228 28074670

[B21] Dittrich A. (1998). The standardized psychometric assessment of altered states of consciousness (ASCs) in humans. Pharmacopsychiatry 31 (Suppl. 2), 80–84. 10.1055/s-2007-979351 9754838

[B22] Doss M. K. Madden M. B. Gaddis A. Nebel M. B. Griffiths R. R. Mathur B. N. (2022). Models of psychedelic drug action: modulation of cortical-subcortical circuits. Brain 145, 441–456. 10.1093/brain/awab406 34897383 PMC9014750

[B23] Farré-Colomés À. Rublinetska O. Soto-Angona Ó. (2026). Psilocybin effects on brain functional connectivity: a systematic review of fMRI studies. Discov. Ment. Health 6, 41. 10.1007/s44192-026-00384-w PMC13003063 41854988

[B24] Fisher R. S. Acharya J. N. Baumer F. M. French J. A. Parisi P. Solodar J. H. (2022). Visually sensitive seizures: an updated review by the Epilepsy Foundation. Epilepsia 63, 739–768. 10.1111/epi.17175 35132632

[B25] Godfrey K. Luan L. X. Timmermann C. (2025). Effects of psychedelics on human oscillatory brain activity. Int. Rev. Neurobiol. 181, 171–202. 10.1016/bs.irn.2025.04.012 40541309

[B26] Gordon N. Hohwy J. Davidson M. J. van Boxtel J. J. A. Tsuchiya N. (2019). From intermodulation components to visual perception and cognition-a review. Neuroimage 199, 480–494. 10.1016/j.neuroimage.2019.06.008 31173903

[B27] Griffiths R. R. Johnson M. W. Carducci M. A. Umbricht A. Richards W. A. Richards B. D. (2016). Psilocybin produces substantial and sustained decreases in depression and anxiety in patients with life-threatening cancer: a randomized double-blind trial. J. Psychopharmacol. 30, 1181–1197. 10.1177/0269881116675513 27909165 PMC5367557

[B28] Gundlach C. Müller M. M. (2013). Perception of illusory contours forms intermodulation responses of steady state visual evoked potentials as a neural signature of spatial integration. Biol. Psychol. 94, 55–60. 10.1016/j.biopsycho.2013.04.014 23665197

[B29] Harari R. Getselter D. Elliott E. (2025). The psychedelic psilocybin and light exposure have similar and synergistic effects on gene expression patterns in the visual cortex. Mol. Brain 18, 23. 10.1186/s13041-025-01191-0 40102929 PMC11921621

[B30] Harding G. F. A. Jeavons P. M. (1994). Photosensitive Epilepsy. Cambridge University Press.

[B31] Hartman A. M. Hollister L. E. (1963). Effect of mescaline, lysergic acid diethylamide and psilocybin on color perception. Psychopharmacologia 4, 441–451. 10.1007/BF00403349 14048565

[B32] Helfrich R. F. Schneider T. R. Rach S. Trautmann-Lengsfeld S. A. Engel A. K. Herrmann C. S. (2014). Entrainment of brain oscillations by transcranial alternating current stimulation. Curr. Biol. 24, 333–339. 10.1016/j.cub.2013.12.041 24461998

[B33] Herrmann C. S. (2001). Human EEG responses to 1-100 Hz flicker: resonance phenomena in visual cortex and their potential correlation to cognitive phenomena. Exp. Brain Res. 137, 346–353. 10.1007/s002210100682 11355381

[B34] Hewitt T. Amaya I. Beauté R. Seth A. K. Schmidt T. T. Schwartzman D. J. (2025). Stroboscopically induced visual hallucinations: historical, phenomenological, and neurobiological perspectives. Neurosci. Conscious 2025, niaf020. 10.1093/nc/niaf020 40799211 PMC12342384

[B35] Kaelen M. Barrett F. S. Roseman L. Lorenz R. Family N. Bolstridge M. (2015). LSD enhances the emotional response to music. Psychopharmacol. Berl. 232, 3607–3614. 10.1007/s00213-015-4014-y 26257162

[B36] Kaelen M. Giribaldi B. Raine J. Evans L. Timmerman C. Rodriguez N. (2018). The hidden therapist: evidence for a central role of music in psychedelic therapy. Psychopharmacology 235, 505–519. 10.1007/s00213-017-4820-5 29396616 PMC5893695

[B37] Kasteleijn-Nolst Trenité D. Rubboli G. Hirsch E. Martins da Silva A. Seri S. Wilkins A. (2012). Methodology of photic stimulation revisited: updated European algorithm for visual stimulation in the EEG laboratory. Epilepsia 53, 16–24. 10.1111/j.1528-1167.2011.03319.x 22091642

[B38] Kometer M. Schmidt A. Jäncke L. Vollenweider F. X. (2013). Activation of serotonin 2A receptors underlies the psilocybin-induced effects on α oscillations, N170 visual-evoked potentials, and visual hallucinations. J. Neurosci. 33, 10544–10551. 10.1523/JNEUROSCI.3007-12.2013 23785166 PMC6618596

[B39] Lefaucheur J.-P. Aleman A. Baeken C. Benninger D. H. Brunelin J. Di Lazzaro V. (2020). Evidence-based guidelines on the therapeutic use of repetitive transcranial magnetic stimulation (rTMS): an update (2014-2018). Clin. Neurophysiol. 131, 474–528. 10.1016/j.clinph.2019.11.002 31901449

[B40] Lumenate Nova (2026). Lumenate. Available online at: https://lumenate.co/lumenate-nova/(Accessed July 16, 2026).

[B41] Maclean K. A. Leoutsakos J.-M. S. Johnson M. W. Griffiths R. R. (2012). Factor analysis of the mystical experience questionnaire: a Study of experiences occasioned by the hallucinogen psilocybin. J. Sci. Study Relig. 51, 721–737. 10.1111/j.1468-5906.2012.01685.x 23316089 PMC3539773

[B42] Madsen M. K. Fisher P. M. Burmester D. Dyssegaard A. Stenbæk D. S. Kristiansen S. (2019). Psychedelic effects of psilocybin correlate with serotonin 2A receptor occupancy and plasma psilocin levels. Neuropsychopharmacology 44, 1328–1334. 10.1038/s41386-019-0324-9 30685771 PMC6785028

[B43] Magni G. Tuena C. Riva G. (2023). A predictive coding approach to psychedelic virtual-induced hallucinations and creative cognition in aging. Front. Hum. Neurosci. 17, 1219052. 10.3389/fnhum.2023.1219052 37484922 PMC10359985

[B44] Marrocu A. Kettner H. Weiss B. Zeifman R. J. Erritzoe D. Carhart-Harris R. L. (2024). Psychiatric risks for worsened mental health after psychedelic use. J. Psychopharmacol. 38, 225–235. 10.1177/02698811241232548 38491857 PMC10944581

[B45] McCulloch D. E.-W. Knudsen G. M. Barrett F. S. Doss M. K. Carhart-Harris R. L. Rosas F. E. (2022). Psychedelic resting-state neuroimaging: a review and perspective on balancing replication and novel analyses. Neurosci. Biobehav Rev. 138, 104689. 10.1016/j.neubiorev.2022.104689 35588933

[B46] Michaiel A. M. Parker P. R. L. Niell C. M. (2019). A hallucinogenic Serotonin-2A receptor agonist reduces visual response gain and alters temporal dynamics in mouse V1. Cell Rep. 26, 3475–3483.e4. 10.1016/j.celrep.2019.02.104 30917304 PMC6559379

[B47] Montgomery C. Amaya I. A. Schmidt T. T. (2024). Flicker light stimulation enhances the emotional response to music: a comparison study to the effects of psychedelics. Front. Psychol. 15, 1325499. 10.3389/fpsyg.2024.1325499 38420171 PMC10901288

[B48] Mortaheb S. Fort L. D. Mason N. L. Mallaroni P. Ramaekers J. G. Demertzi A. (2024). Dynamic functional hyperconnectivity after Psilocybin Intake is primarily associated with Oceanic boundlessness. Biol. Psychiatry Cogn. Neurosci. Neuroimaging 9, 681–692. 10.1016/j.bpsc.2024.04.001 38588855

[B49] Moujaes F. Ji J. L. Rahmati M. Burt J. B. Schleifer C. Adkinson B. D. (2024). Ketamine induces multiple individually distinct whole-brain functional connectivity signatures. Elife 13, e84173. 10.7554/eLife.84173 38629811 PMC11023699

[B50] Muthukumaraswamy S. D. Carhart-Harris R. L. Moran R. J. Brookes M. J. Williams T. M. Errtizoe D. (2013). Broadband cortical desynchronization underlies the human psychedelic state. J. Neurosci. 33, 15171–15183. 10.1523/JNEUROSCI.2063-13.2013 24048847 PMC6618409

[B51] Nichols D. E. (2016). Psychedelics. Pharmacol. Rev. 68, 264–355. 10.1124/pr.115.011478 26841800 PMC4813425

[B52] Notbohm A. Kurths J. Herrmann C. S. (2016). Modification of brain oscillations *via* rhythmic light stimulation provides evidence for entrainment but not for superposition of event-related responses. Front. Hum. Neurosci. 10, 10. 10.3389/fnhum.2016.00010 26869898 PMC4737907

[B53] Preller K. H. Vollenweider F. X. (2018). Phenomenology, structure, and dynamic of psychedelic states. Curr. Top. Behav. Neurosci. 36, 221–256. 10.1007/7854_2016_459 28025814

[B54] Preller K. H. Razi A. Zeidman P. Stämpfli P. Friston K. J. Vollenweider F. X. (2019). Effective connectivity changes in LSD-induced altered states of consciousness in humans. Proc. Natl. Acad. Sci. 116, 2743–2748. 10.1073/pnas.1815129116 30692255 PMC6377471

[B55] Rickli A. Moning O. D. Hoener M. C. Liechti M. E. (2016). Receptor interaction profiles of novel psychoactive tryptamines compared with classic hallucinogens. Eur. Neuropsychopharmacol. 26, 1327–1337. 10.1016/j.euroneuro.2016.05.001 27216487

[B56] Romeo B. Kervadec E. Fauvel B. Strika-Bruneau L. Amirouche A. Verroust V. (2024). Safety and risk assessment of psychedelic psychotherapy: a meta-analysis and systematic review. Psychiatry Res. 335, 115880. 10.1016/j.psychres.2024.115880 38579460

[B57] Schartner M. M. Carhart-Harris R. L. Barrett A. B. Seth A. K. Muthukumaraswamy S. D. (2017). Increased spontaneous MEG signal diversity for psychoactive doses of ketamine, LSD and psilocybin. Sci. Rep. 7, 46421. 10.1038/srep46421 28422113 PMC5396066

[B58] Schwartzman D. J. Schartner M. Ador B. B. Simonelli F. Chang A. Y.-C. Seth A. K. (2019). Increased spontaneous EEG signal diversity during stroboscopically-induced altered states of consciousness bioRxiv [Preprint] 511766. 10.1101/511766

[B59] Stoliker D. Egan G. F. Friston K. J. Razi A. (2022). Neural mechanisms and psychology of psychedelic ego dissolution. Pharmacol. Rev. 74, 876–917. 10.1124/pharmrev.121.000508 36786290

[B60] Stoliker D. Preller K. H. Novelli L. Anticevic A. Egan G. F. Vollenweider F. X. (2025). Neural mechanisms of psychedelic visual imagery. Mol. Psychiatry 30, 1259–1266. 10.1038/s41380-024-02632-3 38862674 PMC11919690

[B61] Strickland J. C. Garcia-Romeu A. Johnson M. W. (2024). The mystical experience questionnaire 4-Item and challenging experience questionnaire 7-Item. Psychedelic Med. 2, 33–43. 10.1089/psymed.2023.0046 40051759 PMC11658653

[B62] Studerus E. Gamma A. Vollenweider F. X. (2010). Psychometric evaluation of the altered states of consciousness rating scale (OAV). PLoS One 5, e12412. 10.1371/journal.pone.0012412 20824211 PMC2930851

[B63] Studerus E. Kometer M. Hasler F. Vollenweider F. X. (2011). “Acute, subacute and long-term subjective effects of psilocybin in healthy humans: a pooled analysis of experimental studies, J. Psychopharmacol. 25, 1434–1452. 10.1177/0269881110382466 20855349

[B64] Studerus E. Gamma A. Kometer M. Vollenweider F. X. (2012). Prediction of psilocybin response in healthy volunteers. PLoS One 7, e30800. 10.1371/journal.pone.0030800 22363492 PMC3281871

[B65] Timmermann C. Roseman L. Haridas S. Rosas F. E. Luan L. Kettner H. (2023). Human brain effects of DMT assessed *via* EEG-fMRI. Proc. Natl. Acad. Sci. U. S. A. 120, e2218949120. 10.1073/pnas.2218949120 36940333 PMC10068756

[B66] Vollenweider F. X. Kometer M. (2010). The neurobiology of psychedelic drugs: implications for the treatment of mood disorders. Nat. Rev. Neurosci. 11, 642–651. 10.1038/nrn2884 20717121

